# Innate immune cells in the pathogenesis of inflammatory bowel disease - from microbial metabolites to immune modulation

**DOI:** 10.3389/fgstr.2024.1452430

**Published:** 2024-12-10

**Authors:** Rabia S. Mousa, Pietro Invernizzi, Hani S. Mousa

**Affiliations:** ^1^ Department of Medicine and Surgery, University of Pavia, Pavia, Italy; ^2^ Division of Gastroenterology, Center for Autoimmune Liver Diseases, European Reference Network on Hepatological Diseases (ERN RARE-LIVER), Istituto di Ricovero e Cura a Carattere Scientifico (IRCCS) Fondazione San Gerardo dei Tintori, Monza, Italy; ^3^ Department of Medicine and Surgery, University of Milano-Bicocca, Monza, Italy; ^4^ School of Clinical Medicine, University of Cambridge, Cambridge, United Kingdom

**Keywords:** IBD - inflammatory bowel disease, macrophages, innate lymphocyte cells (ILCs), short chain fatty acid (SCFA), Crohn’s disease, ulcerative colitis, neutrophils, innate immunity

## Abstract

Inflammatory Bowel Disease (IBD) is a term used to describe a group of disorders characterized by chronic inflammation of the gastrointestinal tract, with Crohn’s Disease (CD) and Ulcerative Colitis (UC) being the most common. While still not fully understood, pathogenesis is believed to be multifactorial – the result of an interplay between genetic susceptibility, immune dysregulation and environmental factors that all lead to chronic inflammation and tissue remodeling. Innate immune cells, which orchestrate the initial defense mechanisms and modulate the subsequent immune response, play a central role in disease initiation and progression. This review examines the complex involvement of innate immune cells in IBD, emphasizing their interactions with environmental factors and the gut microbiome. We highlight the importance of microbial dysbiosis and impaired intestinal barrier function in disease pathogenesis, and the role that innate immune cells play not only as first responders, but also as key players in maintaining intestinal barrier integrity and gut microbiome. This review provides a comprehensive summary of the role that innate immune cells play in IBD pathogenesis with emphasis on the increasingly recognized role of the gut microbiome. A better understanding of innate immune cell mechanisms and of microbiome-immune interactions is key for the development of novel targeted therapies.

## 1 Introduction

Inflammatory Bowel Disease (IBD), which includes Crohn’s Disease (CD) and Ulcerative Colitis (UC), presents a significant medical challenge due to its chronic nature and complex pathogenesis. Characterized by chronic, relapsing inflammation of the gastrointestinal tract, IBD not only disrupts patients’ quality of life but also imposes a significant burden on healthcare systems worldwide. Despite decades of research, the precise etiology of IBD remains elusive. However, it is becoming increasingly clear that the disease emerges from an intricate interplay of genetic susceptibility, environmental triggers, and an aberrant immune response to the gut microbiota.

At the center of this dysregulated immune response lies the innate immune system, the body’s first line of defense against microbial and environmental threats. In a healthy gut, cells such as neutrophils, macrophages, dendritic cells (DCs), and innate lymphoid cells (ILCs) act harmoniously to maintain homeostasis, ensuring the integrity of the intestinal barrier while regulating the delicate balance between tolerance and immunity. However, in IBD, the balance of immune regulation is disrupted, leading to chronic inflammation and tissue damage. Genetic mutations, such as those in the NOD2 gene, impair bacterial sensing and immune activation, while microbial dysbiosis fuels maladaptive immune responses. This cascade of dysregulated signaling pathways perpetuates chronic inflammation, driving tissue damage and disease progression.

Recent advances in our understanding of innate immunity have shed light on the nuanced roles these cells play in both protecting and damaging the gut. Neutrophils, once thought to be simple infantry of the immune system, are now recognized as key regulators of inflammation, capable of both amplifying and resolving immune responses. Macrophages, with their ability to switch between pro-inflammatory and tissue-repairing states, are central to the balance between intestinal damage and healing. Dendritic cells, as the main antigen presenting cells, modulate the crosstalk between innate and adaptive immunity, while ILCs orchestrate mucosal repair and maintain epithelial integrity.

Here, we review the roles of these key innate immune cells in the pathogenesis of IBD, exploring how their dysregulation contributes to disease onset, progression, and resolution. Understanding the molecular mechanisms by which these cells influence the inflammatory milieu provides new opportunities for therapeutic intervention, potentially leading to more targeted and effective treatments.

## 2 Neutrophils

**Figure 1:**
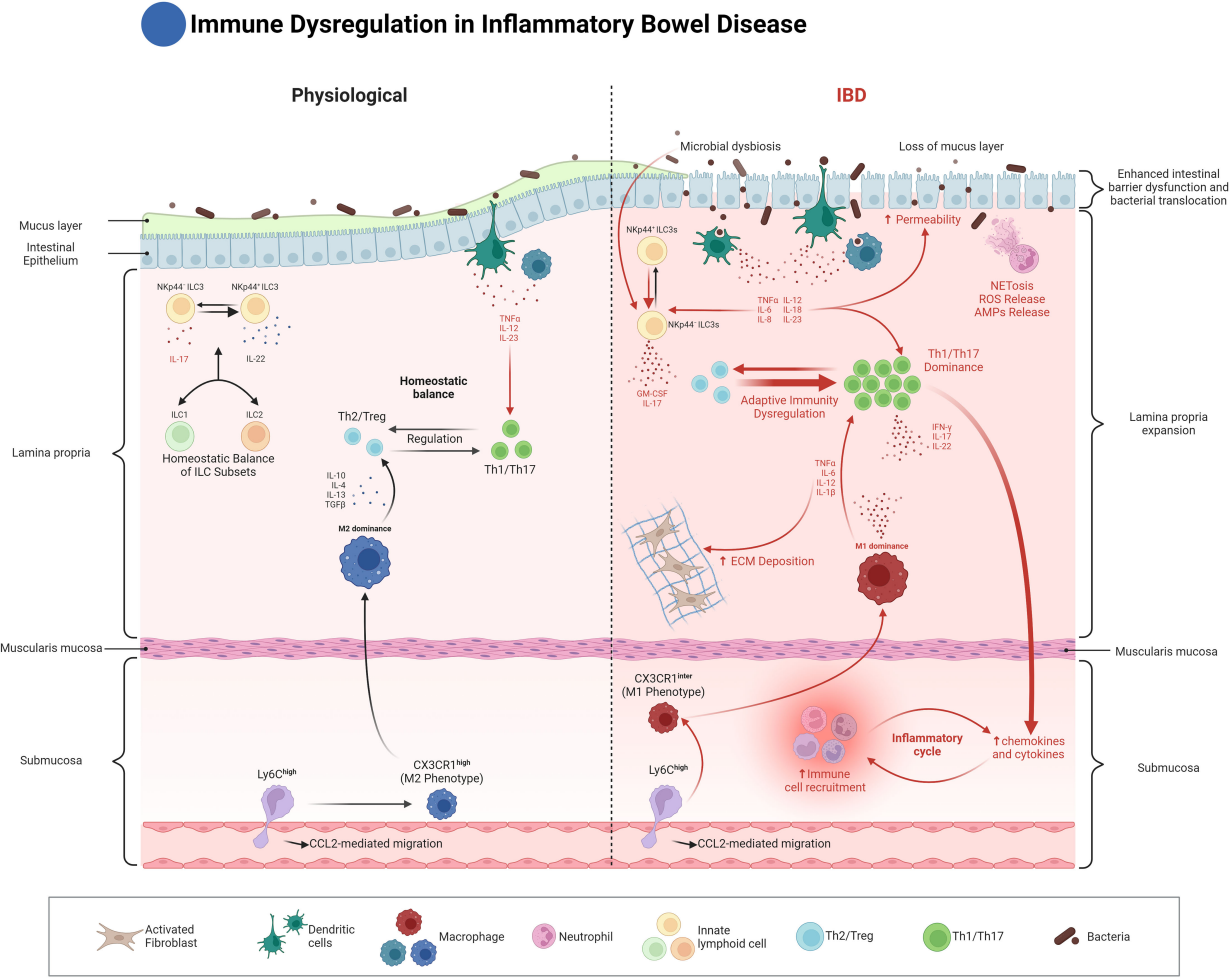
Innate immune dysregulation in Inflammatory Bowel Disease (IBD). This figure compares the immune landscape of normal and inflamed intestines in IBD. On the left, the normal gut is characterized by a balance between regulatory T cells (Th2/Treg) and pro-inflammatory T cells (Th1/Th17), regulated by cytokines like IL-10 and TGF-β. Homeostatic balance of ILC subsets (ILC1, ILC2, and ILC3) is maintained, contributing to epithelial integrity and protection against microbial threats. Dendritic cells and macrophages in the lamina propria play crucial roles in antigen presentation and immune regulation, while Ly6C^high^ monocytes differentiate into tissue-resident macrophages that help maintain gut homeostasis. On the right, the IBD state is characterized by microbial dysbiosis, loss of the mucus layer, and increased intestinal permeability. These changes lead to the dominance of pro-inflammatory Th1/Th17 responses, driven by increased antigen presentation from dysregulated dendritic cells (DCs), along with increased NETosis and ROS production by neutrophils. CX3CR1^Inter^ macrophages adopt an M1 phenotype, driving chronic inflammation, ECM deposition, and fibrosis. Dysregulated ILCs, particularly NKp44- ILC3s, produce elevated levels of IL-17 and IL-22, contributing to further epithelial damage and immune cell recruitment. The figure emphasizes the critical dysregulation of innate immune responses in perpetuating the inflammatory cycle in IBD.

Neutrophils play a dual role in the intestine. Their ability to combat microorganisms, through the release of Neutrophil Extracellular Traps (NETs) and the process of degranulation, is essential for defending the intestinal barrier against pathogens (1, 2). Under physiological conditions, neutrophils undergo programmed cell death (apoptosis) after an inflammatory event and are subsequently cleared by macrophages (efferocytosis), helping to restore tissue balance. In Inflammatory Bowel Disease (IBD), these processes are impaired, contributing significantly to intestinal inflammation, particularly during acute phases and flare-ups. In ulcerative colitis (UC), neutrophils dominate the inflammatory infiltrate in intestinal tissue, and their persistent activation, excessive infiltration, and reduced apoptosis contribute to chronic inflammation (3–8) (**Figure 1**).

Recent studies using single-cell RNA sequencing (scRNA-seq) and spatial transcriptomics have provided unprecedented insight into the complexity and heterogeneity within neutrophil populations in IBD, highlighting their dual roles as both protective and deleterious actors in the disease process. Neutrophils are no longer seen as a homogeneous population with only antimicrobial functions; rather, they display diverse phenotypes and functional states that are influenced by the local tissue environment and inflammation. Three distinct neutrophil subtypes (N1, N2, and N3) have been identified within the inflamed colonic mucosa, each exhibiting unique transcriptional profiles and spatial distribution patterns. N1 and N3 neutrophils share similarities with peripheral blood neutrophils, suggesting they may represent more traditional neutrophil populations. In contrast, N2 neutrophils express high levels of CXCR4 and markers associated with tissue localization and alternative activation states, such as CCL3 and LGALS3. These N2 neutrophils were found to be more abundant in crypt abscesses and ulcerated areas, indicating their potential role in the more severe tissue damage observed in IBD. On the other hand, N3 neutrophils exhibit a pronounced interferon-response signature, reflecting the diverse functional states neutrophils can adopt in response to the inflammatory milieu of the gut (9). This emerging understanding suggests that neutrophils in IBD might adopt distinct activation states, which could be pivotal in shaping disease outcomes. One subset of neutrophils, defined by the expression of CD177 (CD177+ neutrophils), appears to play a unique role in the regulation of IBD. CD177 is a glycoprotein that has been associated with a more functionally activated state, and CD177+ neutrophils have been shown to exhibit enhanced migratory capabilities and increased degranulation, which contributes to their heightened antimicrobial activity. However, in the context of IBD, these neutrophils also seem to exert a protective effect by modulating the inflammatory response through the release of IL-22 and TGF-β. Specifically, CD177+ neutrophils have been found to negatively regulate excessive inflammation in the gut, in part by the interaction of CD177 with PECAM-1, a molecule involved in the transmigration of neutrophils across the endothelial barrier. Through this interaction, CD177+ neutrophils are better able to navigate the inflammatory milieu and resolve inflammation without causing excessive tissue damage (10).

Alongside the heterogeneity of neutrophils, recent studies have highlighted the critical role of Caspase recruitment domain 9 (CARD9), an IBD susceptibility gene, in maintaining proper neutrophil function and protecting against intestinal inflammation. CARD9 is essential for preventing mitochondrial dysfunction in neutrophils, and CARD9 deficiency can lead to increased mitochondrial reactive oxygen species (mtROS) production and premature apoptosis. This mitochondrial overactivation results in reduced neutrophil survival, particularly in oxidative environments, impairing their ability to contain fungal infections and contributing to greater intestinal inflammation (11). In CARD9-deficient neutrophils, the cells exhibit an increased basal activation state, likely due to dysregulated signaling, but despite this heightened activation, they fail to control microbial invaders effectively. This paradox occurs because the neutrophils experience mitochondrial dysfunction, leading to premature apoptosis and reduced functional capacity, impairing their ability to mount a sustained antimicrobial response. As a result, the inability to clear pathogens exacerbates gut inflammation (11–17).

**Figure 2:**
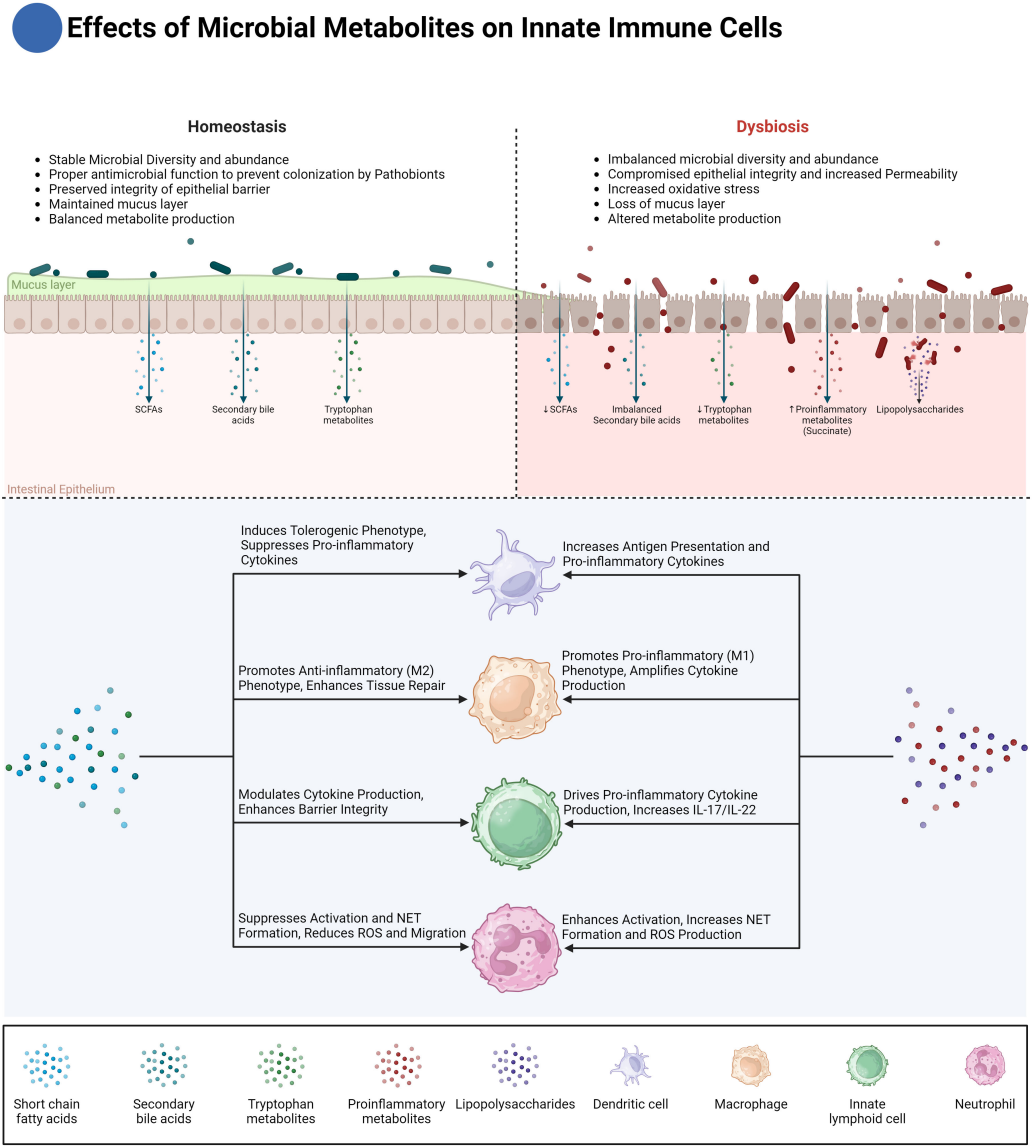
Effects of microbial metabolites on innate immune cells. This figure illustrates the differential effects of microbial metabolites on innate immune cells in homeostasis and dysbiosis. Under homeostasis, microbial diversity and abundance are stable, maintaining a balanced production of metabolites such as short-chain fatty acids (SCFAs), secondary bile acids, and tryptophan metabolites. These metabolites play crucial roles in maintaining epithelial barrier integrity, inducing anti-inflammatory macrophage (M2) phenotypes, and enhancing tissue repair. Dendritic cells (DCs) are modulated to suppress pro-inflammatory responses, while innate lymphoid cells (ILCs) promote barrier integrity through cytokine modulation. Conversely, in dysbiosis, microbial diversity is imbalanced, leading to reduced SCFAs and dysmetabolism of bile acids, which not only results in the accumulation of pro-inflammatory secondary bile acids but also a simultaneous reduction in anti-inflammatory secondary bile acids. Additionally, increased intestinal permeability exposes the immune system to antigens like lipopolysaccharides (LPS), while pro-inflammatory metabolites such as succinate further amplify the inflammatory response. These changes drive pro-inflammatory responses in macrophages (M1 phenotype), amplify cytokine production, and increase neutrophil activation, leading to excessive reactive oxygen species (ROS) production and NET formation. Dysregulated ILC activity promotes the secretion of pro-inflammatory cytokines IL-17 and IL-22, exacerbating tissue damage and inflammation. The diagram also highlights the critical crosstalk between microbial metabolites and immune cells in regulating intestinal inflammation and homeostasis.

Moreover, CARD9 plays a role in sustaining the gut microbiota, where its deficiency has been linked to dysbiosis and increased fungal colonization. This dysbiotic environment can further disrupt the immune response by impairing the production of IL-22, a cytokine crucial for recovery from dextran sulfate sodium (DSS)-induced colitis (18–20). The release of IL-22 is regulated by aryl hydrocarbon receptor (AHR), a transcription factor expressed in intestinal epithelial cells, macrophages, and certain lymphocytes in the gastrointestinal tract. AHR is activated by gut bacteria converting tryptophan into metabolites like indole-3-acetic acid (IAA) and indole-3-aldehyde (IAld) (**Figure 2**). Upon AHR activation, these microbial-derived tryptophan catabolites not only promote IL-22 production but also suppress neutrophil-driven inflammation by inhibiting the release of pro-inflammatory cytokines such as IL-8. Additionally, AHR activation reduces the generation of reactive oxygen species (ROS) in neutrophils, which are central to the inflammatory response and tissue damage. This modulation of neutrophil activity by AHR limits excessive neutrophil influx to inflamed sites, preventing overactivation in response to the gut microbiota and ultimately reducing neutrophil-driven tissue damage in mucosal environments like the gut (18, 20–30). Notably, *Lactobacillus reuteri* has been shown to protect against DSS-induced colitis in mice by generating tryptophan derivatives like indole-3-aldehyde, which in turn activates AHR and promotes further IL-22 production (18, 31–33). These findings suggest that targeting CARD9-related pathways, including mitochondrial function and AHR activation, could potentially be targeted, particularly in patients with CARD9 polymorphisms associated with increased disease susceptibility.

Beyond CARD9, the broader interaction between neutrophils and the intestinal microbiota has also emerged as a critical factor in IBD pathogenesis. Neutrophils influence microbiota composition and function through direct mechanisms, such as phagocytosis and the release of antimicrobial peptides (AMPs) and reactive oxygen species (ROS), as well as indirect mechanisms involving the modulation of the intestinal environment. These interactions are bidirectional, as the microbiota also affects neutrophil function and activation. For instance, dysbiosis, characterized by an overgrowth of pathogenic bacteria like *Enterobacteriaceae*, can exacerbate neutrophil-driven inflammation by providing persistent microbial stimuli that drive continuous neutrophil activation. Conversely, certain commensal bacteria can produce metabolites, such as short-chain fatty acids (SCFAs), that help regulate neutrophil activity, reducing inflammation and promoting tissue repair (34).

In this context, butyrate, a short-chain fatty acid produced by the gut microbiota, has emerged as a significant modulator of neutrophil function (**Figure 2**). Butyrate has been shown to inhibit the production of pro-inflammatory cytokines and chemokines by neutrophils, including IL-6, TNF-α, IFN-γ, and calprotectin, all of which are critical in the pathogenesis of IBD. By suppressing these inflammatory mediators, butyrate helps reduce the overall inflammatory burden in the gut. Furthermore, butyrate also impairs neutrophil migration and NET formation, two key processes that contribute to mucosal damage in IBD. Specifically, butyrate reduces the production of reactive oxygen species (ROS) and myeloperoxidase (MPO), both of which are crucial for NET formation and are implicated in the oxidative damage observed in IBD (35). The ability of butyrate to curtail neutrophil responses underscores its potential as a therapeutic agent for managing mucosal inflammation in IBD. Butyrate’s effects on neutrophils are mediated through multiple pathways. One key mechanism is the inhibition of histone deacetylases (HDACs), which are enzymes that modulate gene expression by altering the acetylation status of histones and other proteins. By inhibiting HDACs, butyrate can downregulate the expression of pro-inflammatory genes in neutrophils and mimicking HDAC inhibition with pharmacological agents can replicate the beneficial effects of butyrate on neutrophils. Interestingly, butyrate’s anti-inflammatory actions do not appear to involve G-protein-coupled receptor (GPCR) signaling, suggesting that butyrate’s modulation of neutrophil function is primarily driven by epigenetic changes rather than receptor-mediated signaling (35).

A key mechanism through which neutrophils contribute to IBD pathology is the formation of NETs, which are composed of DNA fibers, histones, and cytotoxic proteins like myeloperoxidase (MPO) and neutrophil elastase (NE). While NETs serve a protective function by trapping and neutralizing pathogens, their components can also be indiscriminately cytotoxic and pro-inflammatory. In IBD, an increase in NET formation has been observed, correlating with disease activity and contributing to tissue damage. These structures not only exacerbate inflammation but also create a feedback loop that perpetuates the inflammatory process in the gut. Studies have shown that NETs are abundant in the inflamed mucosa of IBD patients, particularly those with active disease, and are associated with key inflammatory markers such as MPO and citrullinated histone H3 (36). Moreover, NETs are not homogenous structures; different stimuli can lead to different types of NET formation, some of which may be more pathogenic than others. For instance, NETs induced by certain bacterial components might differ in composition and pro-inflammatory potential from those induced by cytokines or other immune mediators, suggesting that targeting specific types of NETs might offer a more nuanced therapeutic approach in IBD (36). Beyond their role in inflammation, NETs also contribute to thrombosis, a common complication in IBD. The pro-thrombotic activity of NETs has been linked to their ability to trap platelets and promote coagulation, which can lead to an increased risk of thromboembolic events in IBD patients. Studies have shown that NETs can enhance procoagulant activity and support fibrin formation, highlighting their role in the intersection between inflammation and thrombosis in IBD. This dual role of NETs as both pro-inflammatory and pro-thrombotic mediators underscores the complexity of their function in IBD and suggests that therapeutic strategies targeting NETs could be beneficial not only for controlling inflammation but also for reducing thrombotic risk (36).

A particularly critical aspect of neutrophil involvement in IBD is their interaction with stromal cells, driven by IL-1 signaling, which has been identified as a major factor in determining patient response to therapy. Recent research has revealed that a subset of IBD patients who do not respond to conventional therapies, such as anti-TNF or corticosteroids, exhibit high levels of neutrophil infiltration, coupled with activation of fibroblasts and significant vascular remodeling at sites of deep ulceration. These activated fibroblasts possess neutrophil-chemoattractant properties that are specifically dependent on IL-1 receptor (IL-1R) signaling, rather than TNF signaling. This suggests that the IL-1R pathway plays a dominant role in driving the recruitment and activation of neutrophils in these patients, leading to a more aggressive and therapy-resistant form of the disease (37). In these non-responsive IBD patients, neutrophils are often found in large numbers within ulcerated tissues, where they contribute to ongoing tissue damage and inflammation. This is facilitated by the upregulation of genes encoding CXCR1/CXCR2 ligands, such as CXCL1, CXCL2, and CXCL8, in fibroblasts, which attract neutrophils to the site of injury. The result is a vicious cycle of neutrophil recruitment, tissue destruction, and further inflammation, which is exacerbated by the presence of IL-1β in the ulcer bed. This IL-1-driven stromal-neutrophil interaction is particularly significant because it identifies a distinct pathotype within the heterogeneous landscape of IBD, which may require targeted therapeutic strategies, such as IL-1R blockade, to effectively manage these patients (37). Considering this IL-1-driven interaction, therapies targeting IL-1 signaling, such as Anakinra, an IL-1 receptor antagonist, have been explored. While Anakinra is primarily used for rheumatic conditions, early studies have shown its potential in treating a subset of IBD patients with elevated IL-1β levels, particularly those who are unresponsive to anti-TNF or corticosteroids. These studies suggest that blocking IL-1β may reduce neutrophil infiltration and inflammation in therapy-resistant cases, providing a promising avenue for managing this distinct IBD pathotype (38, 39).

Neutrophils are also key players in the crosstalk between immune cells and epithelial cells during wound repair, which is crucial in the context of IBD. Upon mucosal injury, neutrophils are among the first responders, migrating to the site of damage to combat infection and manage acute inflammation. Once the inflammatory milieu begins to subside, these neutrophils transition into a pro-repair state, releasing a variety of cytokines, chemokines, and growth factors that promote epithelial cell proliferation and migration, facilitating the rapid restoration of the intestinal barrier. Specifically, neutrophil-derived TGF-β has been shown to induce the production of amphiregulin (AREG) by intestinal epithelial cells, which plays a crucial role in promoting epithelial cell differentiation and proliferation, thereby supporting efficient wound healing and maintaining mucosal homeostasis (40).

The therapeutic potential of targeting neutrophils in IBD is further underscored by recent studies investigating the artemisinin analogue SM934. This compound has been shown to ameliorate DSS-induced colitis in mice by suppressing the infiltration and activation of neutrophils and macrophages. Specifically, SM934 reduces the expression of CD11b, a marker associated with the activation of these immune cells, thereby attenuating the inflammatory response in the colon. This reduction in neutrophil and macrophage activity correlates with a decrease in pro-inflammatory mediators such as TNF-α and IL-6, highlighting SM934’s potential as a novel therapeutic agent for managing IBD (41).

In summary, neutrophils in IBD play a complex role, which can be protective as well as pro-inflammatory. Through interactions with the microbiota, CARD9 signaling, NET formation, and involvement in tissue repair, neutrophils are central to both pathogen defense and chronic inflammation (**Figure 1**). Their phenotypic diversity, distinct effector mechanisms and stromal interactions underscore the fine balance they maintain between tissue protection and damage. Therapies targeting neutrophil-driven pathways, like modulating their response with butyrate or inhibiting excessive NET formation, offer a promising potential for managing inflammation in IBD.

## 3 Monocytes and macrophages

In the adult, two distinct populations of macrophages have been described: (1) tissue-resident macrophages that originate from yolk sac-derived erythro-myeloid progenitors and populate tissues during embryogenesis and (2) monocyte-derived macrophages, which differentiate in adulthood from monocytes recruited to sites of injury (42). The phenotype of these monocyte-derived macrophages depends on the local intestinal environment. Intestinal macrophages are distinguished by specific markers, such as CD64 and CX3CR1, which help identify them amidst the diverse immune cell populations in the gut. This accurate identification is crucial, as these macrophages exhibit significant heterogeneity, with subsets expressing markers like CD4 and Tim4, each playing distinct roles in intestinal health and disease (43, 44). Intestinal macrophages require constant replenishment from blood-derived Ly6C^high^ monocytes, which is crucial for maintaining gut homeostasis and for responding to inflammatory conditions (43, 45)​.

Recent research indicates that the traditional classification of these macrophages into M1 or M2 phenotypes, influenced by the surrounding cytokine milieu, is an oversimplification. Instead, macrophages display a spectrum of phenotypes, defined by specific flow cytometric markers and transcriptional signatures, adapting to the tissue-specific microenvironment (46–48).

One end of this spectrum is characterized by macrophages exhibiting functions similar to the traditionally termed ‘M1’ phenotype. These macrophages are induced by factors such as interferon-gamma, GM-CSF, and LPS. They are characterized by the production of pro-inflammatory cytokines, including TNF, IL-1β, IL-12, IL-18, and IL-23, which drive Th1- and Th17-mediated immune responses, crucial in IBD pathogenesis (**Figure 1**).

Conversely, at the other end of the spectrum, macrophages display properties aligning with the ‘M2’ classification. These macrophages emerge in response to stimuli like IL-4, IL-13, M-CSF, glucocorticoids, and TGF-beta. They are associated with tissue repair and the resolution of inflammation. These macrophages are marked by the expression of mannose receptor (CD206) and scavenger-type receptors (CD163 and CD204), secrete IL-10 and TGF-β, and play an important role in regulating immune cell activation (**Figure 1**). IL-10 and its cognate receptor, IL-10R, are pivotal in promoting intestinal homeostasis and have been linked to the development of IBD (49–55). In a murine model of IBD, deficiency of the alpha subunit of Il-10ra in intestinal macrophages leads to the production of IL-23, which in turn triggers the secretion of IL-22 by Th17 cells and ILC3. The released IL-22 then activates intestinal epithelial cells to produce antimicrobial peptides, which inadvertently exacerbate IBD by promoting the recruitment of neutrophils (56). Furthermore, modification of the microbiome by colonization with an IL-10-inducing E. coli strain has been shown to increase the number of IL-10-generating macrophages and alleviate colitis symptoms (57).

An imbalance in these macrophage activities, with a skewing towards what was traditionally termed ‘M1 dominance,’ leads to elevated levels of cytokines like IL-6, IL-23, and TNF-α, increasing cytotoxicity and phagocytic activity. This aspect is particularly significant considering the critical role of TNF and IL-6 in IBD pathogenesis, as underscored by the efficacy of anti-TNF therapies. While the M1/M2 paradigm offers a simplified framework for understanding macrophage function, it fails to capture the complexity seen in the gut. In particular, macrophages in the GI tract exhibit a host of unique phenotypes driven by local cues, illustrating the limitations of this binary classification, especially in the context of IBD.

Monocytes recruited from the bloodstream into the gut undergo a carefully regulated differentiation process. Upon entering the intestinal tissue, Ly6C^high^ monocytes progressively lose Ly6C expression and acquire key macrophage markers such as F4/80, MHCII, and CX3CR1 (43–45). This differentiation cascade, commonly referred to as the “monocyte waterfall,” transforms newly recruited monocytes into tissue-resident macrophages that play essential roles in maintaining gut homeostasis and immune tolerance (43).

Under normal conditions, fully differentiated CX3CR1^high^ macrophages are critical for preserving intestinal barrier integrity. These macrophages extend transepithelial dendrites (TEDs) into the gut lumen, allowing them to sample microbial and food antigens without triggering an inflammatory response (45). Furthermore, they produce anti-inflammatory mediators such as interleukin-10 (IL-10) and help limit excessive immune activation by regulating interactions with T cells. The CX3CR1-CX3CL1 axis is central to maintaining this balance, and disruptions in this pathway can lead to the dysregulation of immune responses and increased susceptibility to inflammatory bowel disease (IBD) (44).

However, during inflammatory conditions such as IBD, the differentiation of Ly6C^high^ monocytes is disrupted. Instead of fully differentiating into CX3CR1^high^ tissue-resident macrophages, many of these monocytes become pro-inflammatory macrophages that express intermediate levels of CX3CR1 and heightened responsiveness to Toll-like receptor (TLR) stimulation (43). The influx of Ly6C^high^ monocytes into inflamed intestinal tissue is driven by chemokine signaling, particularly through the C-C motif chemokine receptor 2 (CCR2), which plays a key role in monocytes recruitment during inflammation (44, 58) (**Figure 1**).

Recent studies have shown that certain therapeutic interventions, such as the use of Vedolizumab, influence not only lymphocyte but also monocyte migration by inhibiting the α4β7 integrin. Non-classical monocytes, which express α4β7 integrin, rely on this pathway for gut-specific homing, especially during intestinal inflammation. Inhibition of α4β7 by Vedolizumab, therefore, indirectly affects the presence of wound-healing macrophages derived from these monocytes. This effect could be a contributing factor to the impaired wound healing and increased rates of postoperative complications observed in some patients undergoing anti-α4β7 therapy for IBD, as fewer monocytes reach the gut tissue to replenish the macrophage pool responsible for resolving inflammation and facilitating tissue repair (59, 60).

Once recruited, Ly6C^high^ monocytes in the inflamed gut express higher levels of TLR2 and NOD2, increasing their sensitivity to microbial stimuli. This heightened sensitivity promotes their differentiation into pro-inflammatory macrophages that secrete mediators like tumor necrosis factor-alpha (TNF-α) and interleukin-6 (IL-6) and express inducible nitric oxide synthase (iNOS), sustaining the inflammatory response. In chronic inflammation, however, macrophages may also adopt pro-fibrotic characteristics, contributing to intestinal fibrosis through pathways that drive excessive extracellular matrix (ECM) deposition (58, 61). This altered differentiation of Ly6C^high^ monocytes into inflammatory and, at times, fibrotic macrophages is crucial to both the inflammatory and fibrotic complications observed in IBD (**Figure 1**).

Thus, the continuum from Ly6C^high^ monocytes to CX3CR1^high^ macrophages represents a critical axis in the maintenance of gut homeostasis and the progression of inflammation in IBD. Under homeostatic conditions, the differentiation of monocytes into CX3CR1^high^ macrophages ensures anti-inflammatory responses and immune tolerance. Conversely, the disruption of this differentiation process during inflammation leads to the accumulation of pro-inflammatory macrophages, exacerbating intestinal damage and contributing to disease pathogenesis (44, 45) (**Figure 1**).

In the dynamic environment of the intestine, macrophage function is not only influenced by their differentiation but also by their ability to adapt metabolically to their surroundings. These metabolic adaptations play a crucial role in the pathophysiology of IBD, as they affect how macrophages respond to inflammatory and homeostatic cues from immune cells, muscle stem cells (MuSCs), and the microbiome.

For instance, pro-inflammatory stimuli such as GM-CSF, secreted by ILC3 cells, induce a metabolic shift in intestinal macrophages towards glycolysis. This shift, driven by upregulation of c-Myc, glucose transporters, and hexokinase 2 (HK2), exemplifies the direct impact of specific cytokines on macrophage metabolism (62–64). Conversely, anti-inflammatory signals such as IL-10 promote a shift towards oxidative phosphorylation (OXPHOS) via the mTORC1 pathway, which plays a crucial role in inflammation control. Disruption of IL-10 signaling, leading to mitochondrial dysfunction, can result in excessive glycolysis and subsequent colitis through NLRP3 inflammasome activation (65).

Macrophage metabolism in IBD is further shaped by interactions with other cell types, such as MuSCs, which secrete IGF2. This growth factor directs macrophages toward an anti-inflammatory phenotype by promoting OXPHOS. The receptor-specific and dose-dependent effects of IGF2 in colitis models underscore its potential as a therapeutic target (66, 67).

In response to inflammatory signals macrophages undergo metabolic reprogramming shifting from OXPHOS to glycolysis. Metabolic intermediates such as itaconate regulate inflammation by modulating key metabolic pathways like glycolysis and the tricarboxylic acid (TCA) cycle (68, 69). These metabolic shifts not only fuel the pro-inflammatory functions of macrophages but also contribute to the epigenetic reprogramming of their activity through processes like histone acetylation, driven by increased acetyl-CoA production (70).

In the context of intestinal inflammation, microbial molecules play a significant role in shaping macrophage metabolism (**Figure 2**). Lipopolysaccharide (LPS), derived from dysbiosis gut bacteria, promotes a pro-inflammatory macrophage phenotype by triggering aerobic glycolysis via TLR4 signaling (71). Moreover, microbial-derived metabolites such as Succinate, a key microbial-derived metabolite, exhibits a dual role in modulating macrophage polarization and inflammation. It promotes pro-inflammatory responses by activating the succinate receptor (SUCNR1), leading to the stabilization of hypoxia-inducible factor 1α (HIF-1α) and subsequent IL-1β production. On the other hand, succinate can also trigger anti-inflammatory effects, particularly in specific contexts such as enhancing type 2 immune responses through intestinal tuft cells and supporting metabolic processes like gluconeogenesis. This balance between pro- and anti-inflammatory outcomes is highly dependent on the local tissue environment and microbial composition (72–75).

In addition to succinate, microbial-derived tryptophan catabolites, particularly indole-3-aldehyde (IAld) and indole-3-propionic acid (IPA), significantly influence macrophage polarization by activating the aryl hydrocarbon receptor (AHR) (**Figure 2**). This AHR activation shifts macrophages toward an anti-inflammatory M2-like phenotype, and enhances the expression of enzymes like heme oxygenase-1 (HO-1), which further contributes to the anti-inflammatory environment by reducing oxidative stress. This shift is crucial for preventing chronic inflammation in the gut, promoting tissue repair, and resolving inflammation once microbial threats are controlled. Additionally, IPA has been shown to strengthen the epithelial barrier by enhancing tight junction proteins, indirectly helping macrophages maintain intestinal barrier homeostasis (18, 26–30, 76, 77).

Beyond tryptophan catabolites, other microbial products, such as short-chain fatty acids (SCFAs) like butyrate which was discussed above, also play a crucial role in regulating macrophage polarization and promoting gut homeostasis (**Figure 2**). Produced by commensal bacteria, butyrate promotes an anti-inflammatory macrophage phenotype by supporting oxidative phosphorylation (OXPHOS) and lipid metabolism. Its interaction with G-protein coupled receptors (e.g., GPR41 and GPR43) and its ability to inhibit histone deacetylases further contributes to maintaining immune homeostasis in the gut (78–84).

In addition, metabolites like secondary bile acids further influence macrophage activity and inflammation in IBD (**Figure 2**). Macrophages, including intestinal macrophages and Kupffer cells in the liver, express bile acid receptors such as GPBAR1 (TGR5) and Farnesoid-X-Receptor (FXR), which are activated by secondary bile acids like lithocholic acid (LCA) and deoxycholic acid (DCA). These receptors play a pivotal role in shaping macrophage polarization and function. Activation of GPBAR1 in macrophages leads to the downregulation of pro-inflammatory cytokines such as TNF-α, IL-6, and IL-1β, promoting an anti-inflammatory phenotype. Similarly, FXR activation helps maintain immune tolerance by modulating macrophage responsiveness to microbial antigens, particularly in the gut-liver axis. This regulatory effect of bile acids on macrophages is essential in maintaining homeostasis and preventing excessive inflammation in conditions like IBD (67, 85–89).

These metabolic shifts, driven by a combination of microbial products, cytokines, and nutrient availability, emphasize the complexity of macrophage function in IBD. The combined influence of pro-inflammatory and anti-inflammatory signals, microbial metabolites, and metabolic reprogramming shapes the inflammatory environment of the gut. By understanding these nuanced metabolic adaptations, including the shifts between glycolysis and oxidative phosphorylation, we can better identify therapeutic targets to regulate macrophage function in IBD. Manipulating pathways such as IL-10 signaling, IGF2-driven OXPHOS, and the effects of microbial metabolites like SCFAs and succinate offers promising avenues for novel treatments.

## 4 Dendritic cells

Dendritic cells (DCs) are pivotal mediators of the immune responses, acting as a bridge between innate and adaptive immunity, and their dysfunction is central to the pathogenesis of Inflammatory Bowel Disease (IBD). They are constantly replenished in gut-associated lymphoid tissues, such as Peyer’s patches and isolated lymphoid follicles, emphasizing their essential role in intestinal immunity (90).

DCs interact with the microbiome to maintain intestinal homeostasis, and disruption of these interactions contribute to chronic inflammation in IBD (91–93).

DCs detect microbial antigens through pattern recognition receptors (PRRs), including Toll-like receptors (TLRs) and C-type lectins, such as mannose receptors (93). Upon recognizing antigens, these receptors initiate signaling cascades that determine DC maturation and their ability to drive T-cell differentiation into Th1, Th2, or regulatory T cells (Tregs) (94). This antigen recognition is crucial for maintaining immune balance, especially in the gut, where constant exposure to microbial stimuli must be tightly regulated to avoid excessive inflammation (**Figure 1**).

There are two major subsets of DCs that play distinct roles in IBD: plasmacytoid DCs (pDCs), identified by high expression of CD123, and conventional/classical DCs (cDCs), marked by CD11c (95–99). Both subsets arise from a common dendritic progenitor (CDP) and are critical in antigen presentation and T-cell activation. Notably, conventional dendritic cell 2 (cDC2) is implicated in driving colitogenic T cells, while conventional dendritic cell 1 (cDC1) may have a protective role (100–103). Under normal conditions, DCs promote tolerance to luminal antigens (104). However, in IBD, they shift towards an inflammatory profile, producing cytokines such as IL-12, IL-6, and IL-18. This inflammatory shift is evident when comparing DC cytokine profiles between healthy individuals and IBD patients (105–108).

Single-cell analyses of IBD patients have further revealed a distinct subset of IL-1β+ dendritic cells, particularly expanded in the mucosa of patients with active Crohn’s disease (CD) and Ulcerative Colitis (UC) (109), that plays a critical role in maintaining chronic inflammation, through IL-1β. Mucosal dendritic cells, including the IL-1β+ subset, exhibit a more inflammatory phenotype compared to peripheral blood DCs, suggesting that local immune responses dictate a phenotype that is fundamentally distinct from that seen in blood (109). These findings indicate that therapies specifically targeting mucosal DCs, particularly the IL-1β+ subset, could be effective in controlling local inflammation and tissue damage in IBD.

In addition to IL-1β, IL-23 is another key cytokine produced by DCs that plays a dual role in IBD. While IL-23 is essential for antimicrobial defense, excessive IL-23 production drives the expansion of Th17 and IFN-γ-producing T cells, contributing to chronic intestinal inflammation (110, 111). Furthermore, miR-10a, highly expressed in intestinal DCs, regulates the IL-12/IL-23 axis by inhibiting the expression of IL-12/IL-23 p40, thus modulating both Th1 and Th17 cell responses that contribute to the inflammatory processes in IBD. This regulation helps control excessive immune activation in the gut, underscoring how disruptions in miR-10a expression can exacerbate cytokine dysregulation and contribute to disease pathogenesis (112). These regulatory mechanisms underscore the complex role of DCs in shaping inflammatory responses in IBD, linking cytokine dysregulation to disease pathogenesis.

Microbiota-derived metabolites are critical modulators of DC function. One of the most well-studied groups of metabolites, short-chain fatty acids (SCFAs), including butyrate and propionate, play a profound role in regulating DC activity within the gut mucosa (**Figure 2**). SCFAs exert their effects on DCs through the inhibition of histone deacetylase (HDAC) activity and the activation of G-protein-coupled receptors (GPCRs) like GPR41, GPR43/FFAR2, and GPR109a (81). Butyrate and propionate have been shown to suppress the activation of bone marrow-derived dendritic cells (BMDCs) by inhibiting lipopolysaccharide (LPS)-induced upregulation of co-stimulatory molecules such as CD40, CD86, and cytokines like IL-6 and IL-12p40. This suppression prevents DCs from adopting a pro-inflammatory phenotype, which is otherwise characteristic of DC dysfunction in IBD. Furthermore, SCFA-exposed DCs are essential in promoting immune tolerance by driving the differentiation of naïve T cells into FoxP3+ Tregs, a critical process for maintaining gut immune homeostasis and preventing excessive immune responses (81).

Dysbiosis in IBD, characterized by a reduction in SCFA-producing bacteria such as *Faecalibacterium prausnitzii* and *Roseburia hominis*, contributes to DC dysfunction. The diminished production of SCFAs results in loss of their protective effects on DCs, promoting a pro-inflammatory microenvironment within the gut. Consequently, therapeutic strategies aimed at restoring SCFA levels, such as butyrate supplementation or the use of high-fiber diets to enhance SCFA production, have been explored as potential interventions for restoring immune balance in IBD (81).

In addition to SCFAs, microbial-derived tryptophan catabolites, such as indole-3-lactic acid (ILA) and indole-3-aldehyde (IAld), also play a role in regulating DC function (**Figure 2**). These catabolites promote the differentiation of tolerogenic DCs via the aryl hydrocarbon receptor (AHR) pathway. When AHR is activated in dendritic cells, it upregulates indoleamine 2,3-dioxygenase (IDO1), creating an immunosuppressive microenvironment. These tolerogenic DCs exhibit decreased expression of co-stimulatory molecules like CD40, CD80, and CD86, while increasing the expression of PD-L1 and TGF-β, promoting the induction of Tregs (26–30). By skewing T cell responses towards tolerance, these DCs prevent excessive inflammation, ensuring that the immune system does not overreact to commensal microbes in the gut.

Similarly, secondary bile acids, such as deoxycholic acid (DCA), have emerged as significant modulators of DC function (**Figure 2**). DCA reduces the expression of co-stimulatory molecules like CD40, CD80, and CD86 on DCs, impairing their ability to activate T cells and skewing the immune response away from pro-inflammatory pathways. Additionally, activation of the farnesoid X receptor (FXR) in DCs promotes the induction of regulatory T cells, further suppressing inflammation (87–89). This highlights another critical mechanism through which microbial metabolites regulate DC activity and maintain gut immune homeostasis.

Therapeutically, SCFA supplementation has shown promise in both experimental and clinical settings. Butyrate enemas have been used as adjuvant therapy for ulcerative colitis, enhancing the efficacy of traditional treatments such as 5-aminosalicylic acid and corticosteroids (81). This evidence suggests that restoring SCFA levels can help modulate DC function, reduce inflammation, and promote immune tolerance, offering a potential therapeutic pathway for managing IBD.

In conclusion, DCs are integral to both maintaining immune tolerance and driving inflammation in IBD. Their dysfunction, influenced by environmental factors such as microbial metabolites, illustrates the complex interplay between innate and adaptive immunity in this disease. A deeper understanding of the role DCs play, especially their interactions with the microbiome and T cells, presents promising avenues for future therapies. Strategies aimed at restoring DC regulatory functions or selectively modulating their pro-inflammatory actions may offer effective treatments for IBD (91, 92, 113).

## 5 Innate lymphoid cells

Although ILCs share developmental origins with lymphocytes, they lack antigen-specific receptors, positioning them as key players in the innate immune system. They are crucial in maintaining the balance between immune activation and tolerance in the gut, playing essential roles in tissue healing and homeostasis (114–118). ILCs originate from a common lymphoid progenitor (CLP) and differentiate into ILC1, ILC2, ILC3, cytotoxic natural killer (NK) cells, and lymphoid tissue inducer (LTi) cells (119–121). Each of these subsets is characterized by distinct functional roles that are essential in gut immunity, particularly in response to microbial and environmental challenges.

The classification of ILCs is based not only on their developmental origin but also on the type of cytokines they produce. Group 1 ILCs, which include NK cells and ILC1s, are known for their production of interferon-gamma (IFN-γ) and tumor necrosis factor (TNF), while NK cells are unique in their potent cytotoxic activity. Group 2 ILCs (ILC2s) play a major role in defending against extracellular parasites and are also involved in allergic inflammation, primarily producing cytokines like IL-5 and IL-13. Additionally, ILC2s are known to secrete amphiregulin (AREG), a key mediator in tissue repair and homeostasis. AREG promotes epithelial cell proliferation and helps maintain epithelial integrity, especially in inflamed tissues such as the gut. In IBD, where prolonged inflammation causes extensive tissue damage, ILC2-derived AREG is crucial in limiting this damage and in promoting recovery (122). Group 3 ILCs (ILC3s) are an important component of mucosal defense, particularly in the gut, producing IL-17 and IL-22 in response to extracellular bacteria and fungi. Similar to Th17 cells, the production of these pro-inflammatory cytokines by ILC3s is regulated by IL-23, and this shared reliance on IL-23, along with their production of IL-17 and IL-22, underscores the functional parallels between Th17 cells and ILC3s in driving inflammation, particularly during active disease states in IBD (123). The ability to tailor immune responses based on specific pathogens highlights the vital role ILCs play in maintaining the delicate immune balance required in the intestinal environment.

The strategic location of ILCs within the intestinal lamina propria places them close to the microbiome, allowing interaction with both symbiotic and pathogenic bacteria. This proximity enables ILCs to regulate inflammation and safeguard the host from microbial threats. In IBD, the ability of ILCs to manage inflammation and prevent tissue damage becomes essential, with shifts in ILC populations observed during active disease phases. For example, in Crohn’s disease, there is an increase in ILC1s, ILC2s, and IL-17-producing ILC3s, along with elevated granulocyte-macrophage colony-stimulating factor (GM-CSF) levels in inflamed tissues. Conversely, a notable decrease in IL-22-producing NKp44+ ILC3s has been reported in the inflamed gut. Although NKp44 is generally considered an activating receptor, aiding in immune defense, its reduced expression in disease correlates with increased disease severity and appears to indicate a shift toward a pro-inflammatory, IFN-γ-driven immune response. This decrease limits the IL-22 production crucial for epithelial barrier maintenance and might result from the inflammatory environment, highlighting the potential regulatory impact of NKp44+ ILC3s in mitigating inflammation and preserving mucosal integrity (114, 124–130) (**Figure 1**).

As previously discussed, short-chain fatty acids (SCFAs) like butyrate are key microbial metabolites in the gut, with significant effects on immune cell function. Beyond their role in dendritic cell modulation, SCFAs also influence other innate immune cells, such as NK cells, a subset of ILCs (**Figure 2**). Butyrate has been shown to play an inhibitory role in regulating NK cell activation and effector functions. Specifically, in NK cells, butyrate significantly reduces the expression of activating receptors, including TRAIL, NKp30, and NKp44, and decreases the production of key pro-inflammatory cytokines, such as IFN-γ and TNF-α. Additionally, butyrate downregulates the cytotoxic activity of NK cells by limiting granzyme B, granzyme A, and perforin production. This inhibitory effect on NK cells is mediated through the suppression of mTORC1 activity, reduction in c-Myc mRNA expression, and alterations in cellular metabolism, ultimately limiting NK cell activation and proliferation (131). The ability of butyrate to modulate NK cell functions may represent a crucial factor in controlling inflammation, preventing excessive immune responses, and maintaining immune homeostasis in the gut.

The effects of other short-chain fatty acids (SCFAs), such as acetate and propionate, have also been implicated in the ILC3-mediated immunomodulation of macrophages. One key mechanism through which they exert their modulation is via the free fatty acid receptor 2 (FFAR2/GPR43), which is expressed on colonic ILC3s and enhances the expression of the transcription factor RORγt. While RORγt is typically associated with Th17 cells, where its activation is often pathogenic in autoimmune diseases, its role in ILC3s is more nuanced. In this context, RORγt is crucial for the production of IL-22, a key cytokine involved in the repair of intestinal epithelial cells (IECs). FFAR2 signaling also promotes the expression of antimicrobial peptides, which are essential for defending against pathogenic bacteria and reducing intestinal inflammation (132–136). Compounds like acetate and propionate, which act as natural FFAR2 ligands, have been shown to increase IL-22 production by ILC3s in mouse models, thereby protecting against experimentally induced colitis (137).

Secondary bile acids like lithocholic acid (LCA) influence the activity of ILCs, especially ILC3s. LCA has been shown to modulate the differentiation of ILC3s via interactions with the vitamin D receptor (VDR), leading to the suppression of IL-17 and IL-22 production (**Figure 2**).

While IL-22 at physiological levels is widely recognized for its role in promoting intestinal epithelial repair and barrier integrity, its effects are highly context-dependent. Under acute inflammatory conditions, IL-22 supports tissue regeneration and microbial defense, protecting the mucosal barrier. However, in the chronic inflammatory environment of IBD, excessive IL-22 can exacerbate inflammation by inducing epithelial cell stress, apoptosis, and the production of inflammatory mediators such as iNOS and TNF-α (138, 139). Elevated IL-22 levels have been associated with the onset of acute colitis and colonic dysplasia in experimental models (140). This paradox is also observed in human IBD, where patients with mild to moderate disease activity exhibit higher levels of IL-22 compared to healthy controls, with these elevations correlating positively with disease activity (141, 142). This highlights that the role of IL-22 in intestinal health and disease is context-dependent, with protective effects at normal levels but potentially pathogenic effects when dysregulated or excessively produced. By modulating IL-22 production, LCA-VDR interactions help maintain a balanced immune environment, potentially limiting chronic inflammation and preserving mucosal integrity in the gut (87–89, 143)​.

As discussed earlier, the aryl hydrocarbon receptor (AHR) is a key regulator of ILC3 function, responding to environmental signals to modulate immune activity. Microbial-derived tryptophan catabolites, such as indole-3-acetic acid (IAA) and indole-3-aldehyde (IAld), regulate ILC3 function by activating AHR (**Figure 2**). AHR activation in ILC3s enhances the production of IL-22, which is essential for maintaining epithelial barrier integrity and promoting the secretion of antimicrobial peptides, such as RegIIIγ and β-defensins. These peptides help protect against microbial invasion and promote mucosal healing, ensuring a balanced immune response in the gut. Furthermore, AHR activation modulates ILC3 responses, preventing excessive inflammation while preserving barrier protection (26–30). Mice deficient in AHR exhibit heightened susceptibility to colitis, which has been attributed to compromised ILC3 function and reduced IL-22 production (22, 144, 145). In these animals, there is also a significant decrease in intraepithelial lymphocytes (IELs), further implicating AHR as a critical factor in maintaining gut barrier integrity (146).

These findings underscore the role of ILC populations, and their cytokine profiles can play in the pathogenesis of IBD. Given the crucial role of the IL-23 pathway in regulating ILC3 function, IL-23 has emerged as a key therapeutic target in IBD. IL-23 inhibitors such as risankizumab and brazikumab have shown promise in controlling inflammation in Crohn’s disease, particularly in patients resistant to anti-TNF therapy (147, 148). Novel therapeutic strategies targeting ILCs are emerging, focusing on the modulation of ILC3s, ILC2s, and their cytokine profiles in relation to the microbiota. These strategies aim to restore immune balance and intestinal barrier integrity in IBD, with particular attention to targeting shared pathways such as the IL-23/Th17 axis (148)​. Future therapeutic strategies might focus on shared pathways between ILC3s and Th17 cells, such as the IL-23 signaling, along with targeting SCFA signaling, FFAR2 modulation, or AHR activation, to restore proper immune regulation and mitigate intestinal inflammation in IBD.

## 6 Discussion

IBD cannot be fully understood without recognizing the central role of the innate immune system in shaping the disease inflammatory landscape. Neutrophils, macrophages, dendritic cells, and innate lymphoid cells (ILCs) are not mere bystanders in IBD; they are pivotal drivers of the immune dysregulation that characterizes this condition. Each of these cell types plays a dual role: they are protectors of the gut but, when dysregulated, they become agents of chronic inflammation and tissue destruction.

Neutrophils, through their ability to form neutrophil extracellular traps (NETs) and release reactive oxygen species (ROS), are critical in both pathogen clearance and tissue injury. Their hyperactivation in IBD highlights the fine line between defense and damage. Macrophages, with their plasticity, have the potential to either drive inflammation or facilitate tissue repair, depending on the signals they receive from their environment. Dendritic cells, which bridge innate and adaptive immunity, play an outsized role in sustaining inflammation through their antigen-presenting capabilities, while ILCs, particularly ILC3s, are emerging as key regulators of epithelial integrity and immune homeostasis (**Figure 1**).

What sets IBD apart from many other inflammatory conditions is the cyclical nature of immune dysregulation and microbial imbalance. Dysbiosis fuels immune activation, which in turn exacerbates microbial disruption, creating a vicious cycle of chronic inflammation (**Figure 2**). Targeting this interplay between the innate immune system and the gut microbiota offers promising therapeutic avenues.

Future therapeutic strategies must focus not only on suppressing inflammation but also on restoring balance within the innate immune system. This includes modulating the activity of hyperactive neutrophils, reprogramming macrophages toward a tissue-repairing phenotype, and restoring the tolerogenic functions of dendritic cells and ILCs. Additionally, therapies aimed at correcting microbial dysbiosis, such as microbiota transplantation or probiotic interventions, could complement immune-targeted treatments and lead to more durable remission.

As our understanding of the molecular and cellular mechanisms driving IBD deepens, it becomes clear that a one-size-fits-all approach is inappropriate. Personalized medicine, guided by genetic, immunologic, and microbiome profiling, holds the key to identifying specific dysregulated pathways in individual patients. This precision approach will enable the development of tailored therapies that not only mitigate inflammation but also restore the integrity of the gut barrier, ultimately leading to more effective and sustained treatments for IBD.

In conclusion, the innate immune system is at the center of IBD pathogenesis and -as such – constitute a promising target for future therapies. By modulating innate immune cells function, we can develop treatments that not only alleviate inflammation but also restore immune balance and promote tissue repair, offering hope for improved outcomes in patients living with this challenging disease.
